# Synergism between x-ray crystallography and NMR residual dipolar couplings in characterizing protein dynamics

**DOI:** 10.1063/4.0000192

**Published:** 2023-07-11

**Authors:** Yang Shen, Ad Bax

**Affiliations:** Laboratory of Chemical Physics, National Institute of Diabetes and Digestive and Kidney Diseases, National Institutes of Health, Bethesda, Maryland 20892, USA

## Abstract

The important role of structural dynamics in protein function is widely recognized. Thermal or B-factors and their anisotropy, seen in x-ray analysis of protein structures, report on the presence of atomic coordinate heterogeneity that can be attributed to motion. However, their quantitative evaluation in terms of protein dynamics by x-ray ensemble refinement remains challenging. NMR spectroscopy provides quantitative information on the amplitudes and time scales of motional processes. Unfortunately, with a few exceptions, the NMR data do not provide direct insights into the atomic details of dynamic trajectories. Residual dipolar couplings, measured by solution NMR, are very precise parameters reporting on the time-averaged bond-vector orientations and may offer the opportunity to derive correctly weighted dynamic ensembles of structures for cases where multiple high-resolution x-ray structures are available. Applications to the SARS-CoV-2 main protease, M^pro^, and ubiquitin highlight this complementarity of NMR and crystallography for quantitative assessment of internal motions.

## INTRODUCTION

The study of protein internal motions at the atomic level has been a topic of high biophysical interest for many decades. These motions are key to the entropic contribution to the free energy function that is of fundamental importance for characterizing protein–protein and protein–ligand interactions.^1,2^ An improved view of protein dynamics may also shed new light on the age-old question of induced fit vs conformational capture.^3–5^

Advances in single particle cryo-electron microscopy have resulted in atomic resolution protein structures, with the heterogeneity in observed structures providing evidence for domain motions that can be of high functional importance.^6,7^ However, at present the atomic details of protein motions and their time scales are more commonly analyzed by protein x-ray crystallography and NMR spectroscopy. In this perspective, we focus on recent innovations in these fields and discuss how their concerted use may lead to further improvements in characterizing the atomic details of molecular motions that drive protein function.

## MOTIONS DERIVED FROM X-RAY CRYSTALLOGRAPHY

Temperature or B-factors contain information on the spatial distribution of dynamic fluctuations experienced by a protein,^8^ but their quantitative interpretation in structural terms is complex.^9–11^ Using molecular dynamics to generate a “protein crystal” perturbed by known multimodal, anharmonic motions, Garcia *et al.* showed that experimental B-factors can substantially underestimate the true mean square displacements.^12^ An analogous, in-depth analysis of simulated data showed that even at high crystallographic resolution, fitted B-factors of some well-resolved atoms can be underestimated by as much as sixfold.^10^ On the other hand, for proteins that diffract to very high crystallographic resolution, electron density often can be confidently interpreted in terms of distinct conformations that are populated in the crystalline state,^13,14^ an observation sometimes referred to as polysterism.^15^ Such structural heterogeneities usually cluster in small regions, typically involving less than half a dozen residues, and the details and relative populations of these conformers vary with temperature.^16^ Residues that exhibit such structural heterogeneities in the crystalline state nearly always give rise to single resonances in solution NMR spectra, recorded at room temperature, indicative of conformational averaging between these substates that is rapid on the NMR timescale of milliseconds.^17,18^ The functional importance of such dynamic interconversions has been well recognized in enzymatic mechanisms,^15,17,19^ and is at the basis of Koshland's induced fit hypothesis which postulated that proteins can switch to alternate discrete conformational states upon ligand binding.^3^

For protein crystals that diffract to high resolution, i.e., better than ∼2-Å, ensemble refinement methods of increasing sophistication have been introduced over the past 30 years.^20–23^ Rather than locally fitting a weighted average of two discrete conformers to the crystallographic data, these methods generate many protein conformers that are sampled from molecular dynamics (MD) trajectories, with these trajectories restrained to yield time-averaged agreement with the x-ray diffraction data.^20^ Such ensemble refinement was shown to be prone to over-fitting of the experimental data, as exemplified by worse cross-validation statistics in terms of the crystallographic free R factor (*R*_free_)^24^ during early applications of this method.^25,26^ However, the introduction of suitable adjustments in both the MD protocol, improved MD force fields, and deformable elastic net (DEN) restraints, mitigate over-fitting and can result in modest improvements in *R*_free_.^21–23^ An intrinsic advantage of ensemble refinement, over the fitting of multiple discrete local conformers, results from the correlation seen in dynamic excursions in different regions of a protein, as expected for example, during allosteric conformational switching. However, it is important to bear in mind that the correlations between motions depend upon the empirical force field used in the MD simulation, and do not result from the Bragg reflections *per se*. Scripts for carrying out crystallographic ensemble refinement using the very powerful and widely used Phenix software^27,28^ have become available, making the approach accessible to non-expert users with sufficiently powerful computational resources.

## DYNAMICS FROM NMR RELAXATION

NMR spectroscopy has long been used for probing the rates and amplitudes of dynamic excursions from the time-averaged structure by measurement of nuclear spin relaxation rates. Motions that are faster than the rotational correlation time of the protein in solution are typically probed by measurement of longitudinal (*R*_1_) and transverse (*R*_2_) relaxation rates of ^15^N or ^13^C nuclei, combined with the magnitude of the heteronuclear nuclear Overhauser enhancement (NOE).^29,30^ The rotational correlation time scales approximately linearly with the molecular volume or mass of the protein, ranging from 4.2 ns for ubiquitin (8.5 kDa) at room temperature, to *ca* 32 ns for hemoglobin (64.5 kDa), and therefore only rather fast motions can be quantitatively probed by such relaxation measurements. The type of motion associated with these relaxation parameters usually cannot be extracted from such data and is simply presented as an effective generalized order parameter, ***S***^2^, that reports on the angular amplitude of the excursions of corresponding bond vectors in a model-free manner.^31–33^ Recent innovative work, relying on transient binding of a protein to slowly tumbling nanoparticles, can extend the timescale over which such dynamic excursions can be probed to values beyond the rotational correlation time of the free protein.^34,35^ Unfortunately, the requirement that the time-weighted average rotational correlation time of the free and nanoparticle-bound protein remains shorter than *ca* 30 ns, the upper limit for which high-resolution amide resonances can be readily observed at adequate sensitivity when using TROSY methods, limits this method to relatively small proteins. Sidechain motions on time scales longer than 30 ns can be studied by the nanoparticle-assisted spin relaxation (NASR) method when using the more favorable methyl group relaxation properties to probe the system.^36^

Internal motions on time scales much slower than the rotational tumbling, in the *μ*s-ms regime, are often reflected in chemical exchange contributions, *R*_ex_, to the transverse relaxation rates, *R*_2_. *R*_ex_ measurement exploits the difference in chemical shifts of the conformationally exchanging substates. For the common situation where the exchange is faster than the difference in chemical shifts (expressed in Hz), the *R*_ex_ contribution to the transverse relaxation rate scales with the square of this shift difference, and inversely with the exchange rate.^37^ In addition to providing precise values of the exchange rate, such measurements also can yield chemical shifts of the substates involved, which contain information regarding the structure of the substate. Although, in practice, it often proves difficult to extract the chemical shifts for more than a single unknown substate, the *R*_ex_ data can be invaluable for validating models of the exchange process that gives rise to the exchange contributions.^38–40^

If precise interproton distances are derived from highly quantitative NOE data, using so-called “exact NOEs” or eNOEs,^41^ regions in a protein structure can sometimes be identified that cannot simultaneously satisfy all precisely measured distances, indicative of switching between conformational substates.^42^ In such cases, multi-conformer ensembles can be derived that show much improved agreement with the NMR data, albeit using an *N*-fold increase in the degrees of freedom when fitting to an ensemble of *N* conformers. Like for crystallographic ensemble refinement, stringent cross-validation criteria are, therefore, needed to prevent over-fitting of the data.^43^ As will be discussed below, residual dipolar couplings (RDCs) provide an unambiguous set of parameters to validate structural models. For example, RDCs that were not used to derive an NMR structure of a small GB3 domain agreed considerably better with a single-conformer eNOE-derived model than with the traditionally NOE-derived structure, validating the power of the eNOE method. However, no significant further improvement was obtained when the structure was refined as a multi-conformer model.^44^

Multi-conformer NMR ensembles also have been generated by using the relaxation-derived backbone order parameters as restraints during MD trajectory calculations.^45^ This dynamic ensemble refinement (DER) method was demonstrated for ubiquitin, showing considerable conformational heterogeneity throughout the structure, much larger than coordinate uncertainties in either the deposited NMR structure,^46^ or calculated from x-ray data by using RAPPER.^47^ However, it is important to note that the original NMR structures of ubiquitin represent best fits to the time-averaged coordinates, whereas the DER method aims to depict the ensemble of structures sampled over time. The DER ensemble showed remarkably good agreement with both scalar couplings and residual dipolar couplings that were not used as restraints during the MD calculation, supporting the methodology. However, we note that ***S***^2^ order parameters, derived from NMR relaxation data, are very sensitive to the N–H bond length used. Due to the low mass of hydrogen, N–H vectors are subject to ultrafast zero-point liberations of rather large amplitudes that decrease the effective ^15^N–^1^H dipolar coupling. Therefore, N–H bond lengths should be artificially lengthened to 1.04 Å when interpreting ^15^N relaxation in terms of motion of the N and C backbone atoms.^48^ The large amplitude dynamics seen in the DER ubiquitin ensemble (PDB entry 1XQQ), forced by imposing compliance with the ^15^N ***S***^2^ values that were derived for a 1.02-Å NH-bond length, resulted in conformers that transiently lack H-bonds (e.g., I30, Q40, L50, and L56), whereas very slow hydrogen exchange^49^ is consistent with their uniform presence in all 190 PDB x-ray structures solved at a resolution ≤2.0 Å. This observation, therefore, suggests that, despite good validation statistics, the DER ensemble representation may be too dynamic.

## DYNAMICS FROM RESIDUAL DIPOLAR COUPLINGS

RDCs contain highly precise information on the time-averaged orientation of bond vectors in the molecular frame of a protein. Measurement of RDCs requires weak alignment of the protein relative to the external magnetic field, either by its own inherent magnetic susceptibility anisotropy^50^ or by the application of an external alignment agent,^51^ most commonly a suspension of filamentous phage^52^ or anisotropically compressed hydrogel.^53,54^

Dipolar coupling between two nuclear spins is described by a rank-2 tensorial interaction, and the averaged RDC for a bond vector that rapidly switches between multiple orientations in the molecular frame therefore will deviate from an RDC calculated for its time-averaged orientation.^55–57^ Depending on the precision at which the experimental RDCs can be measured, such deviations only become detectable for root mean square angular excursions that are larger than *ca* 20°–30°. With the availability of a large set of very precisely measured RDCs and NOEs, ensemble refinement of ubiquitin yielded a small, cross-validated improvement for a two-conformer ensemble over a single conformer, but with local outliers that appeared to be artifactual.^58^

Alternatively, if couplings can be measured under five orthogonal protein alignments (in rank-2 tensor space), these couplings allow the generation of NMR ensemble models that reflect the amplitudes and directions of bond vector fluctuations.^55,59,60^ A landmark study that applied this technology to ubiquitin found evidence for large amplitude backbone dynamics on a microsecond timescale,^59^ termed “recognition dynamics.”^59^ The multi-alignment approach for extracting dynamics hinges on the inability to satisfy the five experimental RDCs, measured for a given bond vector under the five different alignment conditions, by a single vector orientation. However, each RDC also contains a random measurement error, and perfect fitting of five erroneous RDCs is possible due to the additional degrees of freedom that are available when representing the vector by an ensemble, even if its orientation is static. This problem of introducing false motion is aggravated when the alignment orientations are not sufficiently orthogonal to one another, in which case small measurement errors in the RDCs require a wider distribution of bond vectors, i.e., larger amplitudes of motion, to satisfy the data. In practice, application of the approach, therefore, requires RDCs that have been measured at very high precision, in particular when the five protein alignments are insufficiently orthogonal to one another. For ubiquitin, RDCs subsequently measured under a protein orientation that was poorly represented in the original study yielded better fits for most of the protein when using a single, static model rather than the ensemble representation,^61^ suggesting that microsecond motions pertain to a much smaller fraction of the protein than originally reported. We also note that, in practice, five linearly independent alignment orientations are difficult to achieve unless relying on paramagnetic tagging,^62^ which introduces other challenges.

Instead, as discussed below, even if measured in only a single alignment medium, the highly precise structural information contained in RDCs can be used to judge the validity and quality of both x-ray ensemble models and conventionally refined structures. Provided that sufficiently many x-ray structures are available for a given protein, RDCs can also be used to select an ensemble representation of such conventional low-energy structures that agrees better with RDC data than any individual structure.

## RDCS AND CRYSTAL STRUCTURES

Clearly, NMR relaxation data and x-ray crystal structures are highly complementary: the x-ray data yield precise coordinates for low-energy states, with the relaxation data reporting on the rates at which they interconvert if populated in solution and provided that this exchange occurs on a NMR-accessible timescale.^63^ Quantitative information on the number of conformers and their relative populations is often difficult or impossible to extract from such relaxation data. However, as discussed below and recently demonstrated for the SARS-CoV-2 main protease (M^pro^), RDCs hold strong potential to provide this missing information.^64^

Agreement between RDCs and protein atomic coordinates depends primarily on four factors: (1) The precision at which RDCs can be measured relative to the range spanned by the RDCs. (2) The precision at which the atomic coordinates are known, which is correlated with the crystallographic resolution and the R_free_ at which the structure was solved.^65,66^ (3) The accuracy at which hydrogen atom positions can be added to an x-ray structure, which can be challenging for amide groups that sometimes deviate substantially from idealized geometry. (4) True differences between a protein structure in the crystalline and solution states. The latter are often attributed to intermolecular contacts and lattice-packing effects, but well-defined differences between local conformations can also be observed between high-resolution structures determined in the same space group. An example of the latter has been discussed for the orientation of the Thr-198 peptide group in the inter-domain linker adjacent to the P5 ligand-binding pocket of numerous M^pro^ x-ray structures, all in the *C*_121_ space group.^67^

Fits of M^pro^ RDCs to each of 350 x-ray structures available in the PDB showed good agreement for most of the α-helical C-terminal domain as well as part of the N-terminal β-sheet-rich domain, but considerably poorer fits for residues in the intervening half of the protein, including its active site and many of its substrate-binding pockets.^68^ Despite the fact that this homodimeric enzyme is large by NMR standards (68 kDa), which adversely impacts the precision at which RDCs can be measured, the fit of ^15^N-^1^H RDCs to structure was shown to be limited by the accuracy of the coordinates rather than RDC measurement error.^64^ This protein, therefore, presents a good testing ground for evaluating the utility of RDCs to quantify internal dynamics. First, we focus on the application of RDCs to evaluate different x-ray ensemble refinement methods.

## RDC FITS TO X-RAY ENSEMBLE REFINEMENT MODELS

Recently, M^pro^ was studied by various crystallographic ensemble refinement procedures, with mixed results. Whereas the standard ensemble refinement procedure, without DEN restraints, applied to the temperature series of x-ray data (PDB entries 7MHF-7MHK) yielded ensembles (7MHL-7MHQ) that, with one exception (7MHL, 100 K), generated small improvements in *R*_free_,^67^ the dynamic distributions of these ensembles differed considerably.^64^

Agreement with RDCs is commonly expressed as a “quality factor,” *Q*, that effectively represents a weighted root mean square difference between *N* measured (*D*^meas^) and predicted (*D*^pred^) RDCs,

$$Q=\sqrt{\sum_{i=1.N}({D_{i}^{\text{pred}}-D_{i}^{\text{meas}})}^{2}/\left[N\left\{D_{a}^{2}\left(4+3{Rh}^{2}\right)/5\right\}\right]},$$ (1)

with *D_a_* and *Rh* being the alignment strength and rhombicity obtained from the singular value decomposition (SVD) best fit. *Q* factors did not improve for the ensembles over fits to the corresponding traditionally refined x-ray structures.^64^ Importantly, however, for these traditionally refined structures the fits of RDCs to amides that were most disordered in the ensemble refinement were *ca* 50% worse than for the least disordered half of M^pro^ residues. This result indicated that the ensemble refinement correctly identified the residues with poorer coordinate accuracy, but not the atomic details of the coordinate distributions, i.e., the motions.

Application of DEN restraints during ensemble refinement, starting from crystallographic data recorded at very high resolution (PDB entry 7K3T; 1.2 Å),^69^ resulted in moderately (*ca* 10%) improved agreement with RDCs over the conventionally refined two-conformer model.^64^ The results for M^pro^ obtained with the recently introduced ECHT ensemble refinement protocol^22^ have also been reported.^69^ ECHT refinement breaks the disorder down to different size scales and provides a more intuitive view of the internal dynamics.

The ECHT-1 ensemble refinement protocol, applied to the *PDB-REDO* coordinates of the 7K3T M^pro^ structure, yielded a moderately improved correlation with the RDCs, in particular for the more dynamic fraction of residues. This improved correlation indicates that the directions of excursions seen in the ensemble agree with motions sensed by the RDCs, even though *Q* factors remain about 20%–40% worse than for highly refined small proteins such as ubiquitin,^61^ or the third IgG-binding domain of protein G.^70^ Another indication that the x-ray ensemble refinement result remains imperfect is obtained when separately considering the most and least dynamic fraction of amides in the ensembles: if motions are correctly represented in the ensemble, separate RDC fits to the most and least disordered vectors in these ensembles should result in very similar *D*_a_ values. However, a 5%–10% increase in *D*_a_ value for the most disordered half of the residues indicated that the amplitudes of the dynamics assigned to their amides was considerably too large.

## RDC FITS TO ENSEMBLES OF X-RAY STRUCTURES

**FIG. 1.**
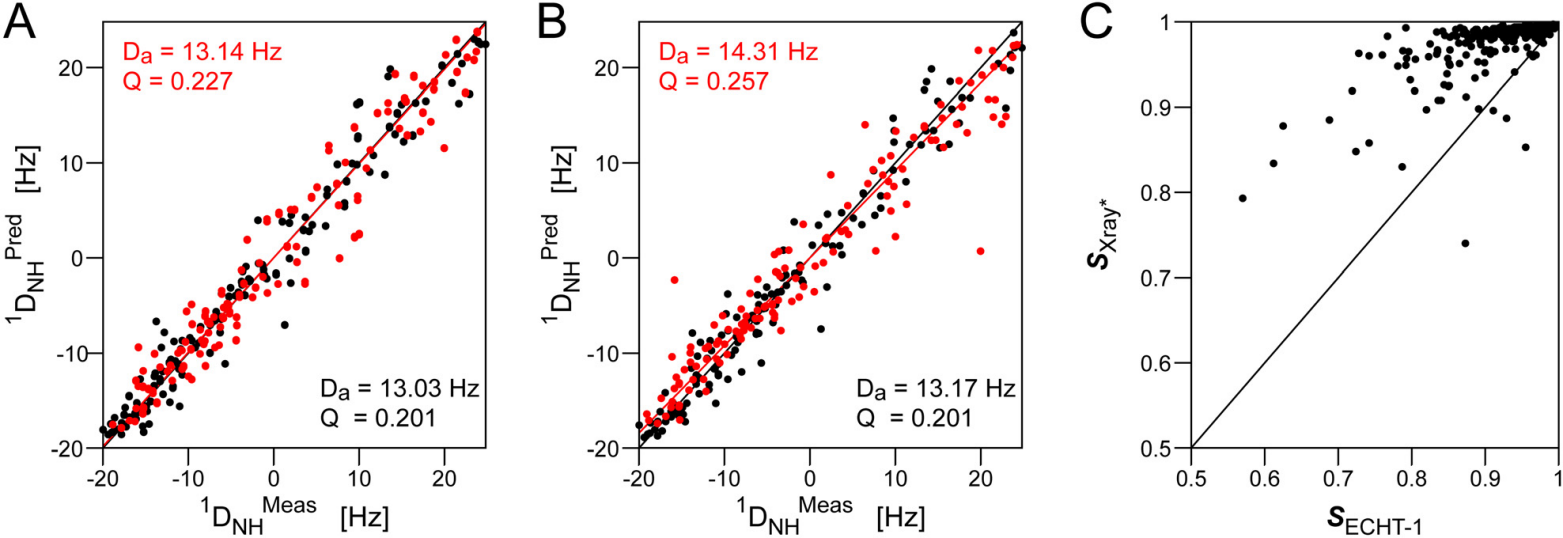
Evaluation of M^pro^ ensembles and their agreement with solution RDCs. (a) Predicted vs measured ^1^D_NH_ RDCs for an ensemble consisting of all 350 SARS-CoV-2 M^pro^ x-ray structures without missing residues, available in the PDB on January 1, 2023. RDCs for the 50% of amides with the smallest (black) and the largest (red) angular excursion yield nearly the same alignment tensors when fit separately to the 350-member ensemble. (b) Analogous correlation for the ECHT-1 ensemble refinement of the 1.2-Å x-ray 7K3T structure by Ploscariu *et al.*^69^ The *D_a_* values best-fitted for the most (red) and least (black) disordered half of residues differ by *ca* 9%. The predicted ^1^D_NH_ RDCs in both (a) and (b) correspond to the *D_a_* values fitted for the most ordered half of the residues. (c) Comparison of backbone N–H generalized order parameters ***S*** calculated from Eq. (2) for the ensemble of 350 x-ray structures (Xray^*^) and the 50-member ensemble obtained by ECHT-1 refinement of 7K3T.

When an artificial “superensemble” was generated from all 350 M^pro^ PDB x-ray structures (distributed over eight space groups, different ligation states, and ranging from 1.2 to 2.85-Å in resolution), considerably better agreement with RDCs was obtained than for any individual structure or any of the crystallographic ensemble refinements (Fig. 1).^64^ Moreover, fits of RDCs to the 50% of residues with the highest disorder (H50%) and lowest disorder (L50%) in this superensemble yielded nearly the same *D*_a_ value,^64^ indicating that the amplitudes of excursions in such an ensemble quantitatively agreed with solution RDCs. For the L50% fraction of residues, the improvement in the ensemble fit over the fit to a single model, ⟨Xray^*^⟩, where each vector adopts the ensemble-averaged vector orientation, is very small (Table I). This result suggests that for these least dynamic residues the improved ensemble RDC fit over the fit to any individual structure is dominated by decreased uncertainty in vector orientations after averaging over 350 different models. In contrast, for the dynamic H50% fraction of residues, the ensemble fit improves by 14% over fitting the single, ensemble-averaged model (Table I), indicating that it is the ensemble distribution, not simply the averaging of uncertainties in orientation, that is responsible for the better fit. For the ensemble fit to the 350 x-ray structures, the *Q* factor for the most dynamic, H50%, residues is only slightly worse than for the L50% half (Table I). This result indicates that this “random collection” of x-ray structures, the vast majority of which were determined by molecular replacement methods but also representing different ligation states and eight space groups, represents a remarkably good quantitative representation of the protein in its unligated solution state that was used for the RDC measurements.

**TABLE I.** Fits of RDCs to M^pro^ backbone amide ^1^H–^15^N and ^1^H–^13^C' vectors for residues with the least (L50%) and most (H50%) disorder in the ensemble of 350 x-ray structures.

		L50%			H50%		
Structure	N^a^	⟨*D_a_*⟩	*Q* (^1^D_HN_)	*Q* (^2^D_C′H_)	⟨*D_a_*⟩	Q (^1^D_HN_)	*Q* (^2^D_C′H_)
Xray^*^^b^	350	13.03	0.201	0.196	13.14	0.227	0.216
⟨Xray^*^⟩^c^	1	12.92	0.204	0.192	12.71	0.264	0.250

^a^ N is the number of conformers used for the RDC fit.

^b^ Xray^*^ is the ensemble of 350 PDB M^pro^ x-ray structures.

^c^ A single-conformer model where N–H and C′–H vectors adopt the ensemble-averaged orientations.

Ubiquitin has also been studied extensively by both NMR and x-ray crystallography. Coordinates for more than 350 ubiquitin chains are available in the PDB, distributed across 31 space groups. Below, we focus on 190 ubiquitin chains that were solved at a resolution ≤2.0 Å. Many of these concern poly-ubiquitin constructs, frequently complexed to other proteins. A large set of highly precise RDCs in solution, measured under four different alignment orientations, is available for free ubiquitin and can be used to evaluate the backbone dynamics of this protein, which often has been used as a benchmark for dynamics analysis by NMR relaxation methods.^34,45,59,71–73^

**FIG. 2.**
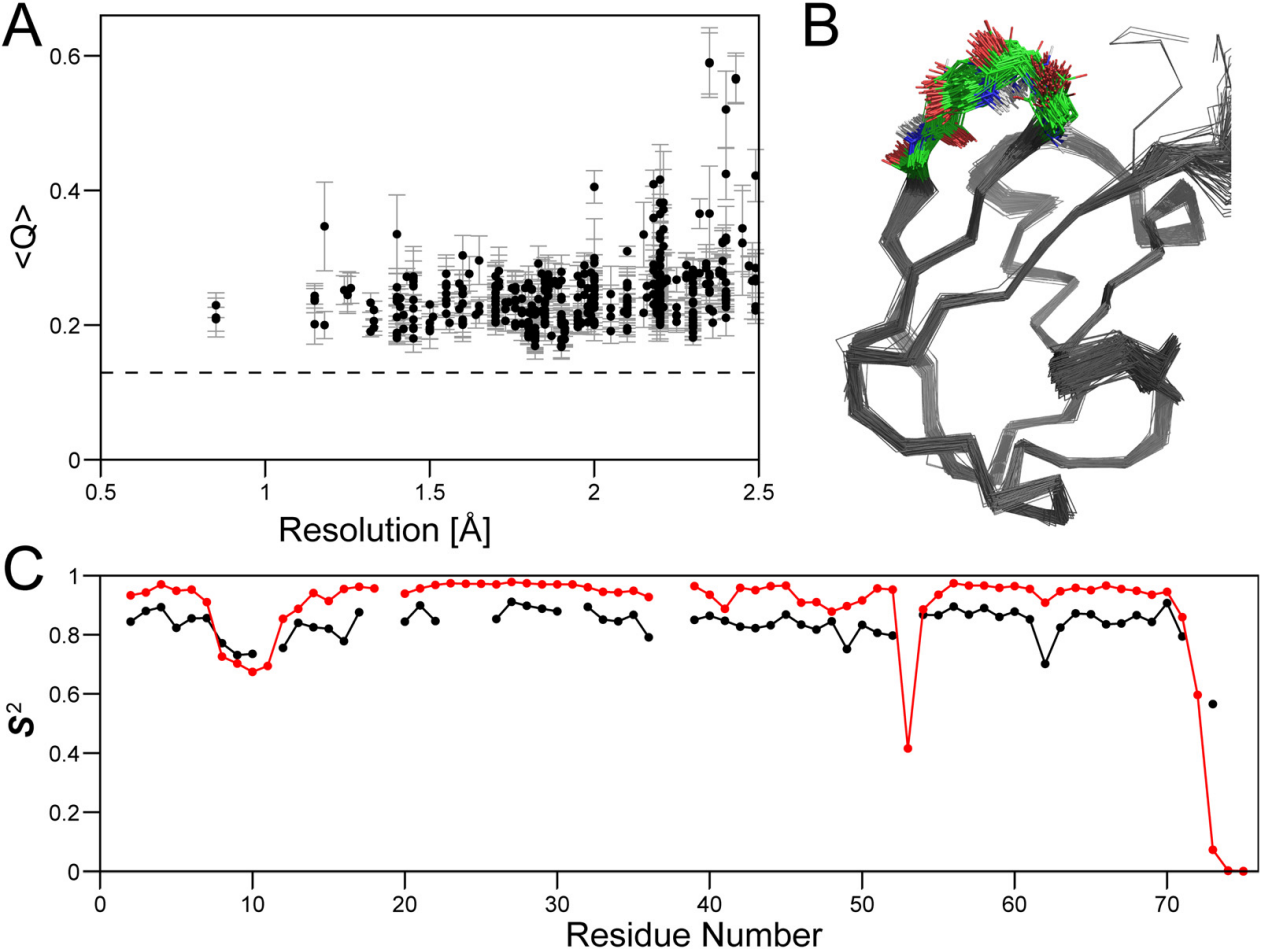
Analysis of PDB ubiquitin x-ray structures (residues Q2-L71). (a) *Q* values of 364 individual chains as a function of crystallographic resolution averaged over ^1^D_NH_, ^1^D_C'N_, ^1^D_C'Cα_, and ^1^D_CαHα_ couplings, recorded in four alignment media.^61^ Error bars correspond to the variation in Q factors over the four alignment media. The dashed line represents the *Q* value (0.129) obtained when fitting the RDCs to an ensemble consisting of all 364 ubiquitin chains in the PDB. (b) Best-fit C^α^ superpositions of residues Q2-L71 for the 190 PDB structures with a resolution ≤2.0 Å, excluding residues L8-T12 (colored) from the best-fit calculation. (c) N–H order parameters, ***S***_NH_^2^, obtained from ^15^N NMR relaxation^72^ (black) and calculated from Eq. (1) (red) for the 190 sets of PDB coordinates to which hydrogens were added using XPLOR-NIH.^74^

A plot of the RDC *Q*-factor against crystallographic resolution confirms that, on average, higher resolution crystal structures agree better with RDCs [Fig. 2(a)]. However, many of the structures solved at above 2-Å resolutions also yield good fits. As was observed for M^pro^, when considering these chains as a dynamic ensemble with equal weights for all members, the fit to the RDCs improves to well beyond what is obtained for any of the individual structures [dashed line in Fig. 2(a)]. A best fit superposition of these chains shows a fairly tight bundle, with a backbone C^α^ coordinate root mean square deviation (rmsd) of 0.65 Å for residues Q2-L71 [Fig. 2(b)].

If the collection of 190 ubiquitin x-ray structures in the Protein Data Bank (PDB) is interpreted as a dynamic ensemble, the N–H angular excursion (β_*n*_) of residue *i* in structure number *n* relative to its ensemble-averaged orientation can be used to generate a generalized order parameter, given by

$$S_{i}=\frac{\left\{3\left\langle {\text{cos}}^{2}\beta_{i,n}\right\rangle -1\right\}}{2},$$ (2)

where the averaging extends over all PDB ubiquitin x-ray structures. The effect of dynamic disorder on NMR relaxation rates scales with ***S***^2^, and it is this squared generalized order parameter that is normally reported in NMR relaxation studies.^29,31^ For regions of secondary structure, these x-ray-derived ***S***^2^ values closely parallel those obtained from NMR relaxation but are higher by ∼0.1 [Fig. 2(c)], consistent with the expected difference from the effect of N–H librations that impact the NMR relaxation analysis (*vide supra*). It is also important to note that, in the absence of slow conformational exchange, the ^15^N NMR relaxation rates are only affected by internal motions that are faster than the overall tumbling time, i.e., *ca* 4 ns for ubiquitin. The lower ***S***^2^ values seen for the L8-T12 loop in the x-ray ensemble indicates that backbone motions in this loop must include rearrangements on a timescale slower than 4 ns, consistent with recent measurements that used nanoparticles to extend the NMR-visible timescale for internal motions.^34^

The high-order parameters seen for most of the protein do not exclude the presence of transient large amplitude motions, such as associated with flipping of aromatic rings or transient unfolding, whose rates are simply too slow to contribute measurably to the ensemble-averaged ***S***^2^ values in relaxation analysis. Similarly, the corresponding high energy states cannot be crystallized and are therefore not observed in the PDB.

## OPTIMIZATION OF THE ENSEMBLE OF STRUCTURES

When multiple x-ray structures for the same protein are present in the PDB, these constitute a rather arbitrary subset of the conformational space sampled by the protein, with each conformer impacted by intermolecular contacts in the crystalline lattice or in molecular complexes that they may be part of. Even if they span the conformational space sampled by the protein in solution, it is highly unlikely that the population of conformers in solution quantitatively corresponds to those deposited in the PDB. Therefore, the above RDC fitting procedure, carried out by assuming equal weights for each of the PDB depositions, is likely to deviate from the true situation and therefore to be sub-optimal. On the other hand, optimizing the weights for each of *N* PDB depositions introduces *N*-1 adjustable parameters in the RDC fit and can result in overfitting. This problem can be mitigated by cross validation, leaving out a small fraction of the RDCs used in the fit, while the remainder is used to optimize the weights and keeping fixed all previously optimized parameters related to alignment tensor orientation and magnitude. Computationally, such cross-validated optimization can become burdensome and improved procedures for ensemble optimization require further development. Efforts at optimizing the weights of the 350 M^pro^ PDB structures, based on a simulated annealing protocol, improved the RDC fit by about 5% over weighing all structures equally.^64^

Another question one may ask is: What is the minimum number of PDB structures needed to fit the RDCs satisfactorily? This latter question becomes of particular interest when focusing on small, local regions with increased dynamics. Can the RDCs of such local regions be satisfied by using a linear combination of just a few structural models? If so, this may yield intuitive insights regarding such motions.

For this purpose, we minimize the function

$$F=\sqrt{\sum_{i=1.N}\sum_{j=P.R}w_{i}({D_{j}^{\text{pred}}-D_{j}^{\text{meas}})}^{2}/\left[(P-R+1)\left\{\frac{D_{a}^{2}\left(4+3{Rh}^{2}\right)}{5}\right\}\right]}-\sum_{i=1.N}\frac{C}{{\left(w_{i}+0.0001\right)}^{2}},$$ (3)

with the restraint ∑_*i=1.N*_ *w*_*i*_ = 1 for the summed weights over *N* structures; *C* is a constant that impacts the weight given to minimizing the number of structures used *vs* minimizing the difference between predicted and measured RDCs, $D_{j}^{\text{pred}}\text{and}\;D_{j}^{\text{meas}}$, for residue *j* for a protein region that spans from residue *P* to *R*; and *D_a_* and *Rh* are the alignment strength and rhombicity, obtained from a global fit of all experimental RDCs to the ensemble of all structures, excluding residues *P* through *R*.

**FIG. 3.**
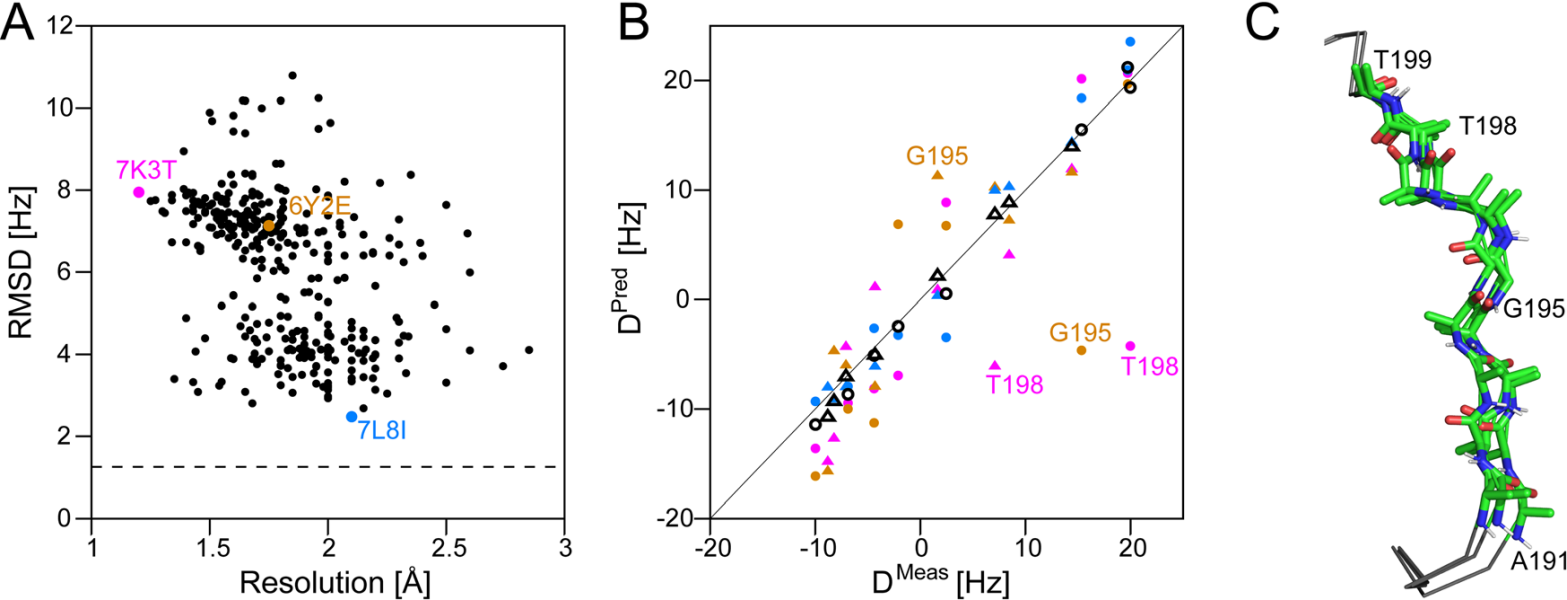
Dynamics in the P5 loop of SARS-CoV-2 M^pro^. (a) RMSD between measured and predicted values for ^1^D_NH_ and ^2^D_C'H_ RDCs of A191-T199, plotted against crystallographic resolution for 350 x-ray structures. ^2^D_C'H_ were upscaled by a factor of 3.09 relative to ^1^D_NH_ values to account for gyromagnetic ratios and internuclear distances, as well as small differences in protein alignment strength,^64^ prior to calculating the RMSD. The dashed line is the RMSD for an optimally weighted 3-member ensemble of PDB entries, marked in color. (b) Correlation between the observed and predicted ^1^D_NH_ (●) and ^2^D_C'H_ (▲) RDCs for A191-T199 in PDB entries 7K3T (lavender), 6Y2E^75^(brown), 7L8I^76^ (blue), and a weighted ensemble (open black) of these three structures populated at 20%, 16%, and 64%, respectively, with the alignment tensor fitted to the backbone amides of residues G2-G302 of 7K3T. (c) Superposition of the A191-T199 linkers of 7K3T, 6Y2E, and 7L8I.

Application of this procedure to the M^pro^ linker, A191-T199, bordering the P5 substrate binding pocket, shows that a satisfactory fit to the data cannot be obtained for any single x-ray structure [Fig. 3(a)]. A fit to all 350 conformers, evenly weighted, agrees better with the RDC data, but not as well as an ensemble of three loop conformers with populations of 20%, 16%, and 64% [Fig. 3(b)]. These three conformers, which represent the minimum needed to satisfy the RDCs to within their experimental uncertainties, exhibit fairly large differences in local structure, providing insights into the type of dynamic excursions at this functional linker site [Fig. 3(b)].

## OUTLOOK

The high degree of complementarity between RDCs and x-ray structures holds strong potential to provide improved, quantitative insights into structural dynamics. To date, the measurement of RDCs for characterizing dynamics has remained a niche, and their analysis in terms of motions remains challenging in the absence of crystallographic data. However, RDC data can rapidly and unambiguously be measured and assigned, and in conjunction with crystallographic data offer new opportunities to characterize protein motions in greater detail.

Recent improvements in crystallographic ensemble refinement, in particular the use of deformable-elastic-net-based restraints, appears to make the procedure more robust.^22^ On the other hand, x-ray ensemble refinements^67,69^ that aimed to describe the internal motions in SARS-CoV-2 M^pro^ only provided a moderate improvement in agreement with RDCs,^64^ measured by NMR for the apo-enzyme in solution. That analysis indicated that the motional amplitudes of the most dynamic regions in the ensemble refinements were substantially overestimated and inconsistent with the RDCs. These findings, therefore, suggest that RDCs can be used as valuable restraints to further optimize the crystallographic ensemble refinement method. However, analysis of M^pro^ also highlighted areas that will remain impossible to capture by crystallographic ensemble refinement. For example, substantial structural differences adjacent to the P5 binding pocket of M^pro^ were seen among different crystal structures, far outside uncertainties related to their electron densities.^67^ Correspondingly, RDCs measured for T198 in this P5 pocket strongly disagree with the highest resolution x-ray structures and one of their ensemble refinements, but agree well with a multi-conformer structure that mostly populates an alternate state seen in several of the other high-resolution structures.

We observed that for two proteins that have been very extensively studied by both x-ray crystallography and NMR, M^pro^ and ubiquitin, improved RDC agreement is obtained for pseudo-ensembles of all PDB x-ray structures over fits to any individual structure. Modest improvement in RDC fits for regions of well-defined secondary structure with below average C^α^ coordinate RMSD appears to be dominated by a more accurate orientation of the bond vectors for the ensemble average over any individual structure, rather than by dynamic effects. However, the substantially larger improvement in pseudo-ensemble RDC agreement seen for the loop regions,^64^ even when compared to ensemble-averaged vector orientations (Table I), indicates that for these more dynamic regions the RDCs contain valuable information on the distribution of conformations sampled by the protein. The improved RDC fits obtained for large ensembles of M^pro^ and ubiquitin x-ray structures suggest that these ensembles of x-ray structures provide fairly realistic descriptions of the conformations sampled in solution. NMR relaxation data then can provide precise time scales for the rates at which such structures interchange, whereas RDCs may aid in adjusting the relative populations.

For both ubiquitin and M^pro^, the large diversity of PDB structures results from the work of dozens of laboratories over multiple years. It appears likely, however, that a much smaller number of structures may suffice to represent the sampled conformational space. At that point, RDCs can be used to judge how representative such a “micro-ensemble” is for its solution structure, and weight factors for its members may be optimized to improve quantitative agreement with the data. Such work will represent an important step toward the ultimate goal of pinpointing the functionally relevant conformers and learning how they interconvert.

## Data Availability

The data that support the findings of this study are available from the corresponding author upon reasonable request.

## References

[1] W. E. Stites , “ Protein-protein interactions: Interface structure, binding thermodynamics, and mutational analysis,” Chem. Rev. 97, 1233–1250 (1997). 10.1021/cr960387h 11851449

[2] R. Perozzo , G. Folkers , and L. Scapozza , “ Thermodynamics of protein-ligand interactions: History, presence, and future aspects,” J. Recept. Signal Transduction 24, 1–52 (2004). 10.1081/RRS-120037896 15344878

[3] D. E. Koshland , “ Application of a theory of enzyme specificity to protein synthesis,” Proc. Natl. Acad. Sci. U. S. A. 44, 98–104 (1958). 10.1073/pnas.44.2.98 16590179 PMC335371

[4] N. Leulliot and G. Varani , “ Current topics in RNA-protein recognition: Control of specificity and biological function through induced fit and conformational capture,” Biochemistry 40, 7947–7956 (2001). 10.1021/bi010680y 11434763

[5] T. Wlodarski and B. Zagrovic , “ Conformational selection and induced fit mechanism underlie specificity in noncovalent interactions with ubiquitin,” Proc. Natl. Acad. Sci. U. S. A. 106, 19346–19351 (2009). 10.1073/pnas.0906966106 19887638 PMC2780739

[6] I. Bahar and A. J. Rader , “ Coarse-grained normal mode analysis in structural biology,” Curr. Opin. Struct. Biol. 15, 586–592 (2005). 10.1016/j.sbi.2005.08.007 16143512 PMC1482533

[7] Y. Yuan *et al.*, “ Cryo-EM structures of MERS-CoV and SARS-CoV spike glycoproteins reveal the dynamic receptor binding domains,” Nat. Commun. 8, 15092 (2017). 10.1038/ncomms15092 28393837 PMC5394239

[8] H. Frauenfelder , G. A. Petsko , and D. Tsernoglou , “ Temperature-dependent X-ray diffraction as a probe of protein structural dynamics,” Nature 280, 558–563 (1979). 10.1038/280558a0 460437

[9] J. Kuriyan , G. A. Petsko , R. M. Levy , and M. Karplus , “ Effect of anisotropy and anharmonicity on protein crystallographic refinement: An evaluation by molecular dynamics,” J. Mol. Biol. 190, 227–254 (1986). 10.1016/0022-2836(86)90295-0 3795269

[10] A. Kuzmanic , N. S. Pannu , and B. Zagrovic , “ X-ray refinement significantly underestimates the level of microscopic heterogeneity in biomolecular crystals,” Nat. Commun. 5, 3220 (2014). 10.1038/ncomms4220 24504120 PMC3926004

[11] Z. T. Sun , Q. Liu , G. Qu , Y. Feng , and M. T. Reetz , “ Utility of B-factors in protein science: Interpreting rigidity, flexibility, and internal motion and engineering thermostability,” Chem. Rev. 119, 1626–1665 (2019). 10.1021/acs.chemrev.8b00290 30698416

[12] A. E. Garcia , J. A. Krumhansl , and H. Frauenfelder , “ Variations on a theme by Debye and Waller: From simple crystals to proteins,” Proteins 29, 153–160 (1997). 10.1002/(SICI)1097-0134(199710)29:2<153::AID-PROT3>3.0.CO;2-E 9329080

[13] H. van den Bedem , A. Dhanik , J. C. Latombe , and A. M. Deacon , “ Modeling discrete heterogeneity in X-ray diffraction data by fitting multi-conformers,” Acta Crystallogr., Sect. D D65, 1107–1117 (2009). 10.1107/S0907444909030613 PMC2756169 19770508

[14] I. W. Davis , W. B. Arendall , D. C. Richardson , and J. S. Richardson , “ The backrub motion: How protein backbone shrugs when a sidechain dances,” Structure 14, 265–274 (2006). 10.1016/j.str.2005.10.007 16472746

[15] J. S. Fraser and C. J. Jackson , “ Mining electron density for functionally relevant protein polysterism in crystal structures,” Cell. Mol. Life Sci. 68, 1829–1841 (2011). 10.1007/s00018-010-0611-4 21190057 PMC3092063

[16] J. S. Fraser *et al.*, “ Accessing protein conformational ensembles using room-temperature X-ray crystallography,” Proc. Natl. Acad. Sci. U. S. A. 108, 16247–16252 (2011). 10.1073/pnas.1111325108 21918110 PMC3182744

[17] J. S. Fraser *et al.*, “ Hidden alternative structures of proline isomerase essential for catalysis,” Nature 462, 669–673 (2009). 10.1038/nature08615 19956261 PMC2805857

[18] R. B. Fenwick , H. van den Bedem , J. S. Fraser , and P. E. Wright , “ Integrated description of protein dynamics from room-temperature X-ray crystallography and NMR,” Proc. Natl. Acad. Sci. U. S. A. 111, E445–E454 (2014). 10.1073/pnas.1323440111 24474795 PMC3910589

[19] R. Otten *et al.*, “ Rescue of conformational dynamics in enzyme catalysis by directed evolution,” Nat. Commun. 9, 1314 (2018). 10.1038/s41467-018-03562-9 29615624 PMC5883053

[20] P. Gros , W. F. Vangunsteren , and W. G. J. Hol , “ Inclusion of thermal motion in crystallographic structures by restrained molecular dynamics,” Science 249, 1149–1152 (1990). 10.1126/science.2396108 2396108

[21] B. T. Burnley , P. V. Afonine , P. D. Adams , and P. Gros , “ Modelling dynamics in protein crystal structures by ensemble refinement,” eLife 1, e00311 (2012). 10.7554/eLife.00311 23251785 PMC3524795

[22] N. M. Pearce and P. Gros , “ A method for intuitively extracting macromolecular dynamics from structural disorder,” Nat. Commun. 12, 5493 (2021). 10.1038/s41467-021-25814-x 34535675 PMC8448762

[23] E. J. Levin , D. A. Kondrashov , G. E. Wesenberg , and G. N. Phillips , “ Ensemble refinement of protein crystal structures: Validation and application,” Structure 15, 1040–1052 (2007). 10.1016/j.str.2007.06.019 17850744 PMC2039884

[24] A. T. Brünger , “ Free *R*-value: a novel statistical quantity for assessing the accuracy of crystal-structures,” Nature 355, 472–475 (1992). 10.1038/355472a0 18481394

[25] F. T. Burling and A. T. Brünger , “ Thermal motion and conformational disorder in protein crystal structures: Comparison of multi-conformer and time-averaging models,” Isr. J. Chem. 34, 165–175 (1994). 10.1002/ijch.199400022

[26] J. B. Clarage and G. N. Phillips, Jr. , “ Cross-validation tests of time-averaged molecular dynamics refinements for determination of protein structures by X-ray crystallography,” Acta Crystallogr. Sect., D 50, 24–36 (1994). 10.1107/S0907444993009515 15299473

[27] P. D. Adams *et al.*, “ PHENIX: Building new software for automated crystallographic structure determination,” Acta Crystallogr. Sect., D 58, 1948–1954 (2002). 10.1107/S0907444902016657 12393927

[28] P. D. Adams *et al.*, “ PHENIX: A comprehensive Python-based system for macromolecular structure solution,” Acta Crystallogr. Sect., D 66, 213–221 (2010). 10.1107/S0907444909052925 20124702 PMC2815670

[29] L. E. Kay , D. A. Torchia , and A. Bax , “ Backbone dynamics of proteins as studied by ^15^N inverse detected heteronuclear NMR spectroscopy: Application to staphylococcal nuclease,” Biochemistry 28, 8972–8979 (1989). 10.1021/bi00449a003 2690953

[30] J. Cavanagh , W. J. Fairbrother , A. G. Palmer , M. Rance , and N. Skelton , *Protein NMR Spectroscopy: Principles and Practice*, 2nd ed. ( Elsevier Academic Press, Burlington, MA, 2007), Chap. 5.

[31] G. Lipari and A. Szabo , “ Model-free approach to the interpretation of nuclear magnetic resonance relaxation in macromolecules. 1. Theory and range of validity,” J. Am. Chem. Soc. 104, 4546–4559 (1982). 10.1021/ja00381a009

[32] G. M. Clore *et al.*, “ Deviations from the simple two-parameter model-free approach to the interpretation of nitrogen-15 nuclear magnetic relaxation of proteins,” J. Am. Chem. Soc. 112, 4989–4991 (1990). 10.1021/ja00168a070

[33] A. G. Palmer , “ NMR characterization of the dynamics of biomacromolecules,” Chem. Rev. 104, 3623–3640 (2004). 10.1021/cr030413t 15303831

[34] S. Wardenfelt *et al.*, “ Broadband dynamics of ubiquitin by anionic and cationic nanoparticle assisted NMR spin relaxation,” Angew. Chem., Int. Ed. 60, 148–152 (2021). 10.1002/anie.202007205 32909358

[35] M. Z. Xie *et al.*, “ Functional protein dynamics on uncharted time scales detected by nanoparticle-assisted NMR spin relaxation,” Sci. Adv. 5, aax5560 (2019). 10.1126/sciadv.aax5560 PMC6693908 31453342

[36] X. Y. Xiang *et al.*, “ Observation of sub-microsecond protein methyl-side chain dynamics by nanoparticle-assisted NMR spin relaxation,” J. Am. Chem. Soc. 143, 13593–13604 (2021). 10.1021/jacs.1c04687 34428032

[37] A. G. Palmer and H. Koss , “ Chemical exchange,” Methods Enzymol. 615, 177–236 (2019). 10.1016/bs.mie.2018.09.028 30638530 PMC7493007

[38] Y. Toyama , R. W. Harkness , and L. E. Kay , “ Structural basis of protein substrate processing by human mitochondrial high-temperature requirement A2 protease,” Proc. Natl. Acad. Sci. U. S. A. 119, e2203172119 (2022). 10.1073/pnas.2203172119 35452308 PMC9170070

[39] M. Tollinger , N. R. Skrynnikov , F. A. A. Mulder , J. D. Forman-Kay , and L. E. Kay , “ Slow dynamics in folded and unfolded states of an SH3 domain,” J. Am. Chem. Soc. 123, 11341–11352 (2001). 10.1021/ja011300z 11707108

[40] A. G. Palmer III , “ Chemical exchange in biomacromolecules: Past, present, and future,” J. Magn. Reson. 241, 3–17 (2014). 10.1016/j.jmr.2014.01.008 24656076 PMC4049312

[41] B. Vogeli *et al.*, “ Exact distances and internal dynamics of perdeuterated ubiquitin from NOE buildups,” J. Am. Chem. Soc. 131, 17215–17225 (2009). 10.1021/ja905366h 19891472

[42] B. Vogeli , S. Kazemi , P. Guntert , and R. Riek , “ Spatial elucidation of motion in proteins by ensemble-based structure calculation using exact NOEs,” Nat. Struct. Mol. Biol. 19, 1053–1058 (2012). 10.1038/nsmb.2355 22940676

[43] B. Vogeli , S. Olsson , P. Guntert , and R. Riek , “ The exact NOE as an alternative in ensemble structure determination,” Biophys. J. 110, 113–126 (2016). 10.1016/j.bpj.2015.11.031 26745415 PMC4806187

[44] B. Vogeli , S. Olsson , R. Riek , and P. Guntert , “ Complementarity and congruence between exact NOEs and traditional NMR probes for spatial decoding of protein dynamics,” J. Struct. Biol. 191, 306–317 (2015). 10.1016/j.jsb.2015.07.008 26206511

[45] K. Lindorff-Larsen , R. B. Best , M. A. DePristo , C. M. Dobson , and M. Vendruscolo , “ Simultaneous determination of protein structure and dynamics,” Nature 433, 128–132 (2005). 10.1038/nature03199 15650731

[46] G. Cornilescu , J. L. Marquardt , M. Ottiger , and A. Bax , “ Validation of protein structure from anisotropic carbonyl chemical shifts in a dilute liquid crystalline phase,” J. Am. Chem. Soc. 120, 6836–6837 (1998). 10.1021/ja9812610

[47] M. A. DePristo , P. I. W. de Bakker , and T. L. Blundell , “ Heterogeneity and inaccuracy in protein structures solved by x-ray crystallography,” Structure 12, 831–838 (2004). 10.1016/j.str.2004.02.031 15130475

[48] M. Ottiger and A. Bax , “ Determination of relative N-H^N^, N-C', C^α^–C', and C^α^–H^α^ effective bond lengths in a protein by NMR in a dilute liquid crystalline phase,” J. Am. Chem. Soc. 120, 12334–12341 (1998). 10.1021/ja9826791

[49] C. Bougault , L. M. Feng , J. Glushka , E. Kupce , and J. H. Prestegard , “ Quantitation of rapid proton-deuteron amide exchange using Hadamard spectroscopy,” J. Biomol. NMR 28, 385–390 (2004). 10.1023/B:JNMR.0000015406.66725.30 14872129

[50] J. R. Tolman , J. M. Flanagan , M. A. Kennedy , and J. H. Prestegard , “ Nuclear magnetic dipole interactions in field-oriented proteins: Information for structure determination in solution,” Proc. Natl. Acad. Sci. U. S. A. 92, 9279–9283 (1995). 10.1073/pnas.92.20.9279 7568117 PMC40968

[51] N. Tjandra and A. Bax , “ Direct measurement of distances and angles in biomolecules by NMR in a dilute liquid crystalline medium,” Science 278, 1111–1114 (1997). 10.1126/science.278.5340.1111 9353189

[52] M. R. Hansen , L. Mueller , and A. Pardi , “ Tunable alignment of macromolecules by filamentous phage yields dipolar coupling interactions,” Nat. Struct. Biol. 5, 1065–1074 (1998). 10.1038/4176 9846877

[53] Y. Ishii , M. A. Markus , and R. Tycko , “ Controlling residual dipolar couplings in high-resolution NMR of proteins by strain induced alignment in a gel,” J. Biomol. NMR 21, 141–151 (2001). 10.1023/A:1012417721455 11727977

[54] H.-J. Sass , G. Musco , S. J. Stahl , P. T. Wingfield , and S. Grzesiek , “ Solution NMR of proteins within polyacrylamide gels: Diffusional properties and residual alignment by mechanical stress or embedding of oriented purple membranes,” J. Biomol. NMR 18, 303–309 (2000). 10.1023/A:1026703605147 11200524

[55] J. R. Tolman , H. M. Al-Hashimi , L. E. Kay , and J. H. Prestegard , “ Structural and dynamic analysis of residual dipolar coupling data for proteins,” J. Am. Chem. Soc. 123, 1416–1424 (2001). 10.1021/ja002500y 11456715

[56] W. Peti , J. Meiler , R. Bruschweiler , and C. Griesinger , “ Model-free analysis of protein backbone motion from residual dipolar couplings,” J. Am. Chem. Soc. 124, 5822–5833 (2002). 10.1021/ja011883c 12010057

[57] S. C. Chiliveri , A. J. Robertson , Y. Shen , D. A. Torchia , and A. Bax , “ Advances in NMR spectroscopy of weakly aligned biomolecular systems,” Chem. Rev. 122, 9307–9330 (2022). 10.1021/acs.chemrev.1c00730 34766756

[58] G. M. Clore and C. D. Schwieters , “ How much backbone motion in ubiquitin is required to account for dipolar coupling data measured in multiple alignment media as assessed by independent cross-validation?,” J. Am. Chem. Soc. 126, 2923–2938 (2004). 10.1021/ja0386804 14995210

[59] O. F. Lange *et al.*, “ Recognition dynamics up to microseconds revealed from an RDC-derived ubiquitin ensemble in solution,” Science 320, 1471–1475 (2008). 10.1126/science.1157092 18556554

[60] J. Meiler , J. J. Prompers , W. Peti , C. Griesinger , and R. Bruschweiler , “ Model-free approach to the dynamic interpretation of residual dipolar couplings in globular proteins,” J. Am. Chem. Soc. 123, 6098–6107 (2001). 10.1021/ja010002z 11414844

[61] A. S. Maltsev , A. Grishaev , J. Roche , M. Zasloff , and A. Bax , “ Improved cross validation of a static ubiquitin structure derived from high precision residual dipolar couplings measured in a drug-based liquid crystalline phase,” J. Am. Chem. Soc. 136, 3752–3755 (2014). 10.1021/ja4132642 24568736 PMC3954408

[62] Y. Wang , L. An , Y. Yang , and L. Yao , “ Generating five independent molecular alignments for simultaneous protein structure and dynamics determination using nuclear magnetic resonance spectroscopy,” Anal. Chem. 92, 15263–15269 (2020). 10.1021/acs.analchem.0c02882 33166130

[63] B. K. Chapman , O. Davulcu , J. J. Skalicky , R. P. Bruschweiler , and M. S. Chapman , “ Parsimony in protein conformational change,” Structure 23, 1190–1198 (2015). 10.1016/j.str.2015.05.011 26095029 PMC4497923

[64] Y. Shen , A. Robertson , and A. Bax , “ Validation of ensemble and static x-ray crystal structure representations of SARS-CoV-2 main protease by solution NMR residual dipolar couplings,” J. Mol. Biol. 435, 168067 (2023). 10.1016/j.jmb.2023.168067 37330294 PMC10270724

[65] A. Bax , “ Weak alignment offers new NMR opportunities to study protein structure and dynamics,” Protein Sci. 12, 1–16 (2003). 10.1110/ps.0233303 12493823 PMC2312400

[66] K. Chen and N. Tjandra , “ The use of residual dipolar coupling in studying proteins by NMR,” in *NMR of Proteins and Small Biomolecules*, edited by G. Zhu (Springer, 2012), Vol. 326, pp. 47–67. 10.1007/128_2011_215 PMC4153736 21952837

[67] A. Ebrahim *et al.*, “ The temperature-dependent conformational ensemble of SARS-CoV-2 main protease (M^pro^),” IUCrJ 9, 682–694 (2022). 10.1107/S2052252522007497 PMC9438506 36071812

[68] A. J. Robertson , J. M. Courtney , Y. Shen , J. F. Ying , and A. Bax , “ Concordance of X-ray and AlphaFold2 models of SARS-CoV-2 main protease with residual dipolar couplings measured in solution,” J. Am. Chem. Soc. 143, 19306–19310 (2021). 10.1021/jacs.1c10588 34757725 PMC8592127

[69] N. Ploscariu , T. Burnley , P. Gros , and N. M. Pearce , “ Improving sampling of crystallographic disorder in ensemble refinement,” Acta Crystallogr. Sect., D 77, 1357–1364 (2021). 10.1107/S2059798321010044 PMC8561732 34726164

[70] T. S. Ulmer , B. E. Ramirez , F. Delaglio , and A. Bax , “ Evaluation of backbone proton positions and dynamics in a small protein by liquid crystal NMR spectroscopy,” J. Am. Chem. Soc. 125, 9179–9191 (2003). 10.1021/ja0350684 15369375

[71] D. M. Schneider , M. J. Dellwo , and A. J. Wand , “ Fast internal main-chain dynamics of human ubiquitin,” Biochemistry 31, 3645–3652 (1992). 10.1021/bi00129a013 1314645

[72] N. Tjandra , S. E. Feller , R. W. Pastor , and A. Bax , “ Rotational diffusion anisotropy of human ubiquitin from ^15^N NMR relaxation,” J. Am. Chem. Soc. 117, 12562–12566 (1995). 10.1021/ja00155a020

[73] D. Fushman , N. Tjandra , and D. Cowburn , “ An approach to direct determination of protein dynamics from ^15^N NMR relaxation at multiple fields, independent of variable ^15^N chemical shift anisotropy and chemical exchange contributions,” J. Am. Chem. Soc. 121, 8577–8582 (1999). 10.1021/ja9904991

[74] C. D. Schwieters , J. J. Kuszewski , and G. M. Clore , “ Using Xplor-NIH for NMR molecular structure determination,” Prog. Nucl. Magn. Reson. Spectrosc. 48, 47–62 (2006). 10.1016/j.pnmrs.2005.10.001

[75] L. Zhang *et al.*, “ Crystal structure of SARS-CoV-2 main protease provides a basis for design of improved α-ketoamide inhibitors,” Science 368, 409–412 (2020). 10.1126/science.abb3405 32198291 PMC7164518

[76] G. J. Lockbaum *et al.*, “ Pan-3C protease inhibitor rupintrivir binds SARS-CoV-2 main protease in a unique binding mode,” Biochemistry 60, 2925–2931 (2021). 10.1021/acs.biochem.1c00414 34506130 PMC8457326

